# Hemocytes and fat body cells, the only professional immune cell types in *Drosophila*, show strikingly different responses to systemic infections

**DOI:** 10.3389/fimmu.2022.1040510

**Published:** 2022-11-23

**Authors:** Vaibhvi Vaibhvi, Sven Künzel, Thomas Roeder

**Affiliations:** ^1^ Department of Molecular Physiology, Zoology Institute, Kiel University, Kiel, Germany; ^2^ Department of Evolutionary Genetics, Max Planck Institute for Evolutionary Biology, Plön, Germany; ^3^ German Center for Lung Research, Airway Research Center North, Kiel, Germany

**Keywords:** hemocytes, fat body, insect immunity, innate immunity, Drosophila immunity, transcriptome

## Abstract

The fruit fly *Drosophila* is an excellent model to study the response of different immunocompetent organs during systemic infection. In the present study, we intended to test the hypothesis that the only professional immune organs of the fly, the fat body and hemocytes, show substantial similarities in their responses to systemic infection. However, comprehensive transcriptome analysis of isolated organs revealed highly divergent transcript signatures, with the few commonly regulated genes encoding mainly classical immune effectors from the antimicrobial peptide family. The fat body and the hemocytes each have specific reactions that are not present in the other organ. Fat body-specific responses comprised those enabling an improved peptide synthesis and export. This reaction is accompanied by transcriptomic shifts enabling the use of the energy resources of the fat body more efficiently. Hemocytes, on the other hand, showed enhanced signatures related to phagocytosis. Comparing immune-induced signatures of both cell types with those of whole-body responses showed only a minimal correspondence, mostly restricted again to antimicrobial peptide genes. In summary, the two major immunocompetent cell types of *Drosophila* show highly specific responses to infection, which are closely linked to the primary function of the respective organ in the landscape of the systemic immune response.

## Introduction

Insects, like all other invertebrates, rely exclusively on the innate immune system in their daily fight against infections of all kinds. However, as they can successfully fight off the vast majority of infections, they offer an impressive example of the efficiency of innate immunity (1). The reduced complexity of the innate compared to the adaptive immune system, however, does not mean that the innate immune system has a simple architecture. This complexity of the innate immune system manifests itself at different scales (2). For example, the immune responses of the fruit fly, as the insect model organism, differ significantly when it is infected with different types of pathogens, such as bacteria, fungi, parasites, or viruses (3, 4). Another level of complexity results therefrom that different organ systems are involved in the immune response. Here, especially the mucosal epithelia on the one hand and the professional immune organs on the other hand must be distinguished from each other. The epithelial immune response, which is particularly operative at the mucosal barrier organs, intestine, and trachea, shows peculiarities that are not found in this way in the other immunocompetent organs of *Drosophila* (5–9).

When it comes to fighting pathogens that have managed to overcome the epithelial surfaces and thus invade the body, two professional immune organs that combat such infections are of particular importance, the fat body, and the hemocytes. The fat body has the task to enable a humoral immunity, i.e. to release antibacterial agents into the hemolymph and therewith fight invading pathogens (1). The hemocytes, on the other hand, are responsible for cellular defense, which requires direct contact between the pathogen and these cells of the immune system to perform the task of pathogen control (10). As already indicated, the fat body and the hemocytes are the two organ systems that professionally deal with pathogen recognition and pathogen defense, which means that they are largely dedicated to pathogen defense. In the case of epithelia, be it the tracheal or the intestinal epithelium, it is assumed that immune defense is only one of several tasks to be performed (6, 11, 12). However, the same may be true for the fat body, which is the fly’s most important storage organ and plays a central role in the control of metabolism (13–15). Nevertheless, it is undisputed that both organs have by far the most important defense functions against invading pathogens. Looking at the molecular mechanisms of pathogen recognition, the central first step of activation of the innate immune system, fat body, and hemocytes show extensive similarities. Both organs use the Toll as well as the IMD signaling pathway for this task (1, 16).

This complexity of the innate immune system of *Drosophila* may be a major reason for the differential immune responses following infections with diverse pathogens (17). A recent study examined a range of bacterial pathogens to identify response specificity, the diversity of the immune reaction, and common signatures representing a “core” immune reaction (17). Interestingly this list of genes differed significantly from genes identified by an earlier transcriptome study as the basal immune-stress induced genes, which were called ‘*Drosophila* Immune Regulatory Genes’ (18). In addition to the immediate immune processes, which primarily involve the production of antimicrobial agents, these studies found activation of metabolic changes, stress responses, and other processes affected by bacterial infection (17, 18).

Despite the large body of information regarding the specificity and similarities of an immune response, critical knowledge gaps still exist in understanding the *Drosophila* immune response. In this context, the crucial question of the contributions of the major immunocompetent organs to the overall immune response remains mostly unanswered. We conducted the described study to fill this knowledge gap and to test the hypothesis that the reaction of both professional immune organs shows a high degree of commonalities. With this aim, we chose a pathogen that induces an intermediate response. Infection with *Bacillus subtilis* was particularly suitable for this purpose, as it showed the required characteristics, the induction of a strong systemic immune response, and the possibility that the fly resolved the infection within a few days. Contrary to our working hypothesis, the immune responses of hemocytes and fat body to infection with *Bacillus subtilis* showed minimal similarities in their transcript signatures, which were essentially limited to the family of antimicrobial peptide genes. Comparison of the immune responses of both organs with the ‘core’ immune response of the whole body also showed very little agreement. In summary, our study shows that the information from whole-animal analyses provides relatively little information about the processes in the major immune organs of the fly.

## Material and methods

### Fly strains and rearing

For all the experiments with tissue collection for sequencing, the strain *w^1118^* (Bloomington Stock Center #5905) was used. Other strains used include: Drs-GFP (Bloomington Stock Center #55707), Hml (Δ)-Gal4;UAS-eGFP (Bloomington Stock Center #30142) and *srpHemo-3xmCherry* (Gift from Daria Siekhaus, IST Klosterneuburg, Austria)). Animals were reared as previously described (19). In detail, they were raised on a standard cornmeal yeast medium at 25°C with 65% relative humidity and 12h:12h L:D cycle. The pupae were transferred to a modified exome matched holidic diet (20) and kept for 12 days on this before the tissues were obtained for sequencing.

### Bacterial infections and sterile injury

The following strains of bacteria were used: *Bacillus subtilis* (ATCC6051), *Serratia marcescens Db11*, *Erwinia carotovora carotovora* (Ecc2046). Infections were performed using secondary cultures of the bacteria grown in LB media at 30°C. The bacteria were suspended in sterile PBS and adjusted to the following OD_600_ for the different bacteria: *S. marscescens* - 0.01, *Ecc2046* – 15, and *B. subtilis* - 1.5. Infections were performed by dipping a minutin needle in this bacterial solution and piercing the thorax of animals (21). For sterile injury, the needle was dipped in sterile PBS and used to pierce the thorax. Bacterial load was also measured using the same protocol (21). Briefly, one fly was taken per sample at the given time, homogenized in PBS, and dot-plated in serial dilutions. The colonies were counted after overnight growth.

### Fat body and hemocyte isolation

Fat body was dissected from the dorsal abdominal region and directly transferred to RNA magic as described earlier (22). Hemocytes were extracted by removing the antenna and applying light pressure on the thorax of the animals to get a clear drop of hemolymph, which was further collected using a microcapillary (23) and directly suspended into RNA magic. For each replicate of fat body, one female was used, while for hemocytes, one replicate was made by pooling material from 5 females.

### Imaging of fat body and hemocytes

Fat bodies were dissected from the control and bacteria infected individuals as described in section 4.3. The tissue was further fixed for 15 minutes in 4% paraformaldehyde at room temperature and mounted on a microscope slide using RotiMount Flour-Care DAPI (#HP20.1, Carl Roth GmbH, Karlsruhe, Baden-Württemberg, Germany). The hemocytes were collected using a microcapillary as described earlier and the hemolymph volume was released onto a microscope slide coated with Poly-L-Lysine. This was immediately followed by putting a small drop of PBS over the hemolymph and incubating the slide in a moist chamber for 15 minutes, to let the cells adhere to the slide. The cells were further fixed using 4% Paraformaldehyde for 10 minutes, gently washed with 0.1% PBST (PBS containing 0.1% Tween 20) three times and mounted using Rotimount. Imaging was performed using fluorescence microscope equipped with Apotome or by using a CLSM880 (Carl Zeiss Image Axio Vision, Jena, Germany).

### RNA extraction and amplification

The cells in RNA Magic were homogenized using a bead ruptor (BioLab Products, Bebensee, Germany), and total RNA was isolated using the phenol-chloroform-isopropanol method. cDNA synthesis and amplification were done using the two-step Smart-Seq3 protocol (24). The amplification was performed using LA Taq TaKaRa (TaKaRa Bio, Shiga, Japan) using their provided protocol. The number of cycles required were tested for each sample by visualising the amplified product on agarose gel with amplification at multiple cycle numbers at a gap of three and picking the point in the exponential phase and before saturation.

### Sequencing and analysis

Library preparation was done using an adaption of the protocol described in Picelli et al., 2014 (25) and sequencing was done on the NextSeq 500 (Illumina, California, US) using the High Output Kit v2.5 (75 cycles). The quality of the reads was checked using fastqc (https://www.bioinformatics.babraham.ac.uk/projects/fastqc/). The trimming of low-quality reads, mapping to the reference genome (BDGP6) and calculation of read counts for each gene were done using CLC workbench (Qiagen, Hilden, Germany). The differential expression of each gene was calculated using the BioConductor package DESeq2 (26) [employing the shrinkage estimator apeglm (27)] and GO term analysis on differential expressed genes was performed using DAVID (28) and flyenrichr (29). Additional graphs were plotted using GraphPad Prism v7. For comparisons of the whole-body data set (17) to our dataset, their read counts for individual replicates were reanalysed using the same procedure as mentioned above. The expression levels for core genes were calculated as the average of the gene expression in all the bacterial treatments.

### Real-time PCR

The amplified cDNA was obtained as mentioned above and used for qRT-PCR following a protocol described earlier (30). Four to five biological replicates (with two technical replicates for each) were taken for every treatment of both cell types. The data was analyzed using ΔΔCt method, using RpL13 as the housekeeper for fat bodies and RpL32 for hemocyte samples. Multiple t-tests were performed to test for statistical significance and the two-stage linear step-up procedure (Benjamini, Krieger and Yekutieli) was used to calculate the adjusted p-value. The primers are mentioned below:

**Table:** 

Primers	Forward (5’-3’)	Reverse (5’-3’)
Rpl32	CCAGTCGGATCGATATGCTAA	GTTCGATCCGTAACCGATGT
Rpl13	CAGGCATGCGAACCGCGTCAA	CAGGCATGCGAACCGCGTCAA
Dip	GATGGTTTTGGCTTTGCAGT	TTTCCAGCTCGGTTCTGAGT
AttB	ACAATCTGGATGCCAAGGTC	TGTCCGTTGATGTGGGAGTA
Dro	GTTCACCATCGTTTTCCTGC	GGCAGCTTGAGTCAGGTGAT
Drs	GTACTTGTTCGCCCTCTTCG	AGCATCCTTCGCACCAGCAC
Mtk	CCACCGAGCTAAGATGCAA	AATAAATTGGACCCGGTCTTG
CecC	AAGATCTTCGTTTTCGTCGC	GTTGCGCAATTCCCAGTC

## Results

### Experimental setup and quality control

**Figure 1:**
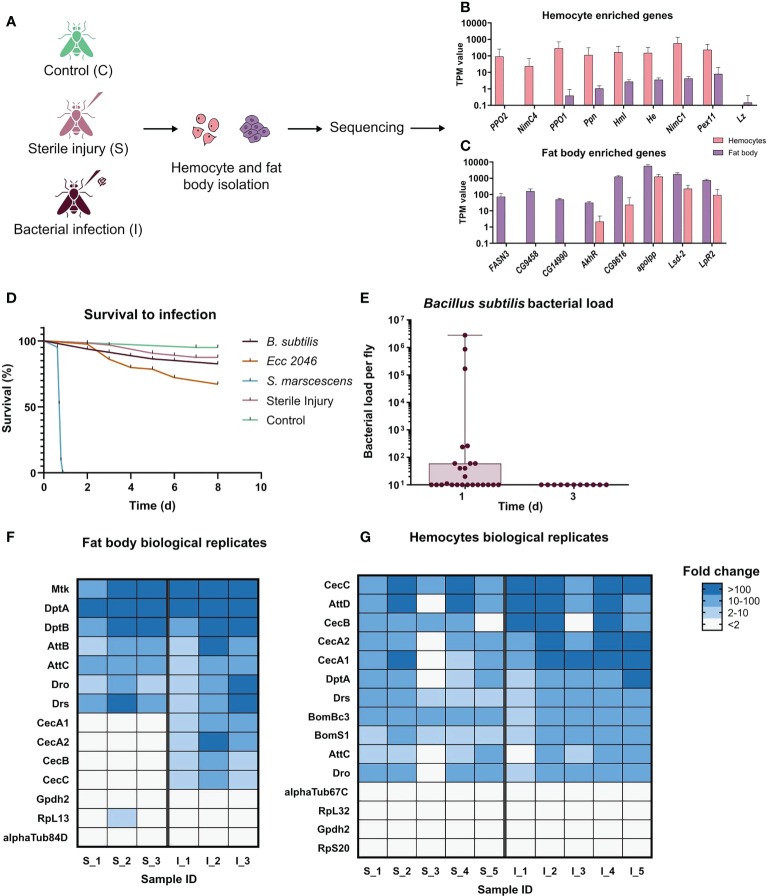
Experimental design of the systemic infection experiments. **(A)** Scheme showing the setup of the experiment. Samples were collected from control(C), sterile injury(S), and bacteria-infected (I) animals. Infections were performed using the bacterium *Bacillus subtilis*. Hemocytes and fat bodies were isolated 20 hours after infection, subjected to RNA extraction, cDNA synthesis, and amplification, and then sequenced. Three biological replicates were sequenced for fat body (one animal per replicate) samples and five for hemocytes (hemolymph pooled from 5 animals in each replicate). **(B)** TPM values for some known hemocyte-enriched genes in both hemocytes and fat bodies samples. Most genes show more than 100-fold higher transcript numbers in the hemocyte samples, indicative for a highly pure hemocyte preparation. **(C)** TPM values for some of the most enriched genes in the adult fat body according to Flyatlas2 (32) in both hemocytes and fat body samples. All genes show much higher expression in the fat body samples, indicative for a highly pure fat body cell preparation. **(D)** The survival curve of flies subjected to the following treatments: Untreated Control (n=40), Sterile Injury (n=64), *S. marcescens* systemic infection (OD_600_ 0.01; n=30), *Ecc2046* systemic infection (OD_600_ 15; n=82), *Bacillus subtilis* systemic infection (n=81). **(E)** Bacterial load of the flies 24 hours (n=25) and 72 hours (n=11) after infection with *Bacillus subtilis*. Each dot represents one animal, mean values ± S.D. **(F)** Heatmap of fold changes of read numbers for selected AMP and housekeeping genes in individual biological replicates for the injured and infected animals’ fat bodies. A number at the end of the treatment name indicates the individual replicates: S_1, S_2 and S_3 are sterile injury and I_1, I_2 and I_3 are bacteria infected. Fold change was calculated relative to the average TPM value in the control fat body. **(G)** Heatmap of fold changes for selected AMP and housekeeping genes in individual biological replicates for the injured and infected animals’ hemocytes. A number at the end of the treatment name indicates the individual replicates: S_1 - S_5 are sterile injury and I_1 - I_5 are bacteria infected. Fold change was calculated relative to the average TPM value in the control hemocytes.

To compare the transcriptome responses of the fat body and hemocytes, we subjected both tissue types to the identical sequencing protocol. However, because adult hemocytes cannot be isolated in such quantities in pure form to allow direct sequencing, we used an amplification protocol for both tissues. Moreover, we employed a cDNA synthesis protocol that accounts for low abundance and hence results in minimal loss (24) and amplified this material by Rapid amplification of cDNA ends PCR [RACE - PCR (31)] (**Figure 1A**) (24).

We confirmed the purity of our hemocyte samples by looking at the hemocyte population obtained using the reporter lines *Hml-GFP* and *srpHemo-mcherry* and observed fluorescence in all cells obtained. Furthermore, we validated our amplification system by comparing the expression of well-known hemocyte marker genes in the hemocyte as well as fat body transcriptomes to confirm their enrichment in the former. These markers included *hemolectin*, *hemese* (33), which are known for all developmental stages, and some larval hemocyte markers including *Prophenoloxidase1*, *Prophenoloxidase2*, *papilin*, *Nimrod C4*, *Nimrod C1* and *Peroxin 11*, which are also present in adult hemocytes (34) (**Figure 1B**). Consistent with the previous larval and adult hemocyte studies, no expression of *glial cells missing* or *glial cells missing 2*, which are only expressed in the embryonic stage of hemocytes (33) or *lozenge*, which is required for crystal cell differentiation, could be detected (**Figure 1B**). Though there is no known marker for crystal cells in adults, the absence of *Lozenge* can indicate that our hemocyte population mainly consists of plasmatocytes. Similarly, for the fat body, the purity of samples was confirmed by the expression of some of the most enriched genes in this tissue according to flyatlas2 including *Fatty acid synthase 3*, *apolipophorin, CG9458, CG14990* and *CG9616* (**Figure 1C**). Additional highly enriched genes in the fat body with known functions in the tissue like *Adipokinetic hormone Receptor, Lipid storage droplet-2 and Lipophorin receptor 2*, were also detected in high abundances (**Figure 1C**) (32).

To identify the most suitable bacterium that generates a systemic infection and a corresponding response from the host, but at the same time causes low lethality and can be eliminated from the host after a few days, we performed preliminary tests with different bacteria. Here, *Bacillus subtilis*, *Erwinia carotovora (Ecc 2046)*, *Serratia marcescens*, and sterile injury was tested (**Figure 1D**). In contrast to infection with *Serratia marcescens* and *Erwinia carotovora (Ecc 2046)*, infection with *Bacillus subtilis* met the expectations and it could be completely eradicated within three days after infection was initiated (**Figure 1E**), implying that *Bacillus subtilis* only has a reduced pathological potential. To validate the functioning of the amplification and sequencing setup for the different treatments, we compared the expression values of a few antimicrobial peptides in the injured and infected animals’ fat body and hemocytes (**Figures 1F, G**). We compared the fold change in the TPM values of these AMPs in individual biological replicates relative to the average values for the respective tissue controls. All biological replicates show a consistent upregulation of most of the AMPs for both the cell types indicating the replicability in the data and showing that our infection induces a robust immune response in each of the biological replicates (**Figures 1F, G**). We additionally included the same analysis for a few housekeeping genes, which showed a maintenance of the expression values in almost all biological replicates. To provide an independent proof of functionality of the setup, we performed qRT-PCR on the amplified cDNA from the different treatments for selected AMPs (**Supplementary Figures 1A, B**). For both the cell types, we observed a significant increase in the expression levels of all the genes upon infection and for some of them upon injury. These results were consistent with what we observed from the transcriptomic analysis.

### Infection induced transcriptional changes in fat body are distinct from whole body responses

**Figure 2:**
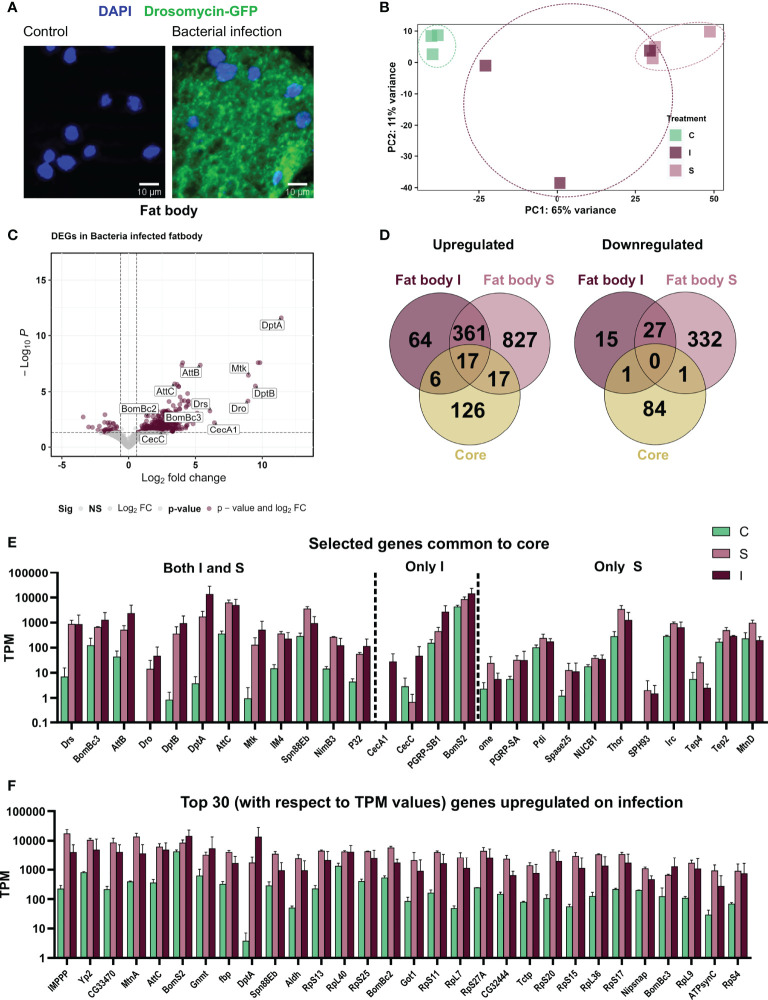
Systemic infection with *Bacillus subtilis* as well as sterile injury exert an immune response in the adult fat body. **(A)** The fat body from the reporter line *Drosomycin-GFP* in non-treated controls (left) and after infection with *B. subtilis* (right). The nuclei are stained with DAPI. **(B)** Principal Component Analysis plot showing the first two principal components for the RNA-seq analysis of control **(C)**, sterile injury (S), and bacteria-infected **(I)** fat bodies. **(C)** Volcano plot depicting the differentially expressed genes in bacteria-infected fat body cells as compared to untreated control fat bodies. The genes passing the threshold (adjusted p-value < 0.05, log_2_fold change > 0.6) were considered differentially expressed and are marked in maroon and the rest of the genes are marked in gray. **(D)** Venn diagram analysis of the common number of regulated genes in the bacteria infected **(I)** and sterile injury (S) fat body data sets and the ‘core’ genes from whole body infection experiments are shown separately for the upregulated and downregulated genes. ***(*E*)*** Transcript per Million (TPM) values are shown for selected genes that were commonly regulated between the full body ‘core’ dataset and the bacteria infected **(I)** and sterile injury (S) fat body data set. **(F)** TPM values are shown for the top 30 upregulated genes (in terms of read counts) in bacteria-infected fat bodies.

To demonstrate that the experimental infection elicited an immune response, we used a reporter line, where expression of GFP is under the transcriptional control of the *drosomycin* promotor (32, 35). Here, strong GFP signals were seen in fat body cells after infection, whereas no signal was seen in non-treated controls (**Figure 2A**). To evaluate the fat body dataset, we first performed a principal component analysis for the three treatment groups (control – C, sterile injury – S, and Infection - I) (**Figure 2B**) and observed very distinct separation and low variance in the samples of control and sterile injury. In contrast, bacteria-infected samples show very high variability. We also observe this high inter-replicate variability in bacteria-infected fat bodies in other experiments (unpublished work), which implies that this variance is an inherent feature of this complex biological treatment (36). To test the validity of our sequencing strategy, we checked the expression of AMP genes known to be regulated in response to infection in infected fat bodies compared to non-infected controls and detected strong increases between isolated fat bodies from infected animals and matching controls. Here, a clear increase in expression of these AMP genes was seen (**Figure 2C**). Comparing the data of infected animals and those subjected to sterile injury with matching controls, we found 490 genes regulated in bacterial infection (447 upregulated and 43 downregulated) and 1580 regulated genes in response to sterile injury (1221 upregulated, 359 downregulated, **Figure 2D**; **Supplemental Table 1**).

**Figure 3:**
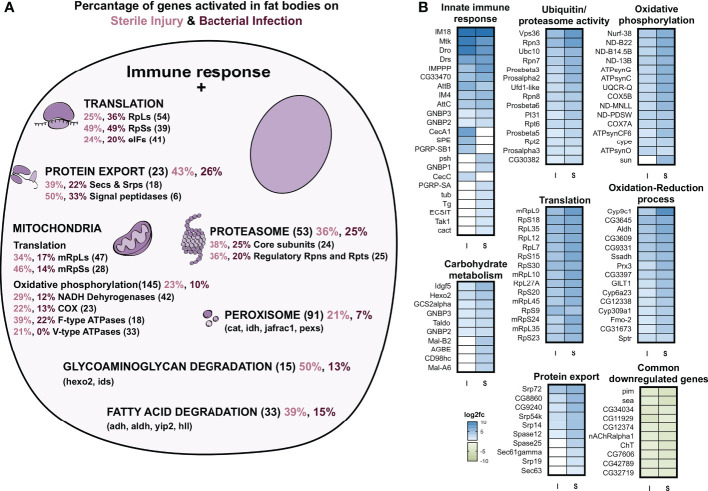
GO term analyses of genes regulated in the fat body in response to infection and sterile injury. **(A)** Summary of the processes activated on top of the classic antibacterial response in fat body cells on bacterial infection or sterile injury. For each process, the total number of genes associated with the process is shown in brackets next to it. Out of these genes, the percent activated in bacterial infection are shown in maroon and in sterile injury in pink color. For some processes, the subunits are shown (like the *RpLs* and *RpSs* for translation) and for some processes, some selected genes are shown (like *hexo2* and *ids* for glycosaminoglycan degradation). **(B)** The log_2_ fold changes of selected genes from the processes shown in A are plotted as heatmaps for bacterial infection (I) and sterile injury(S). For the innate immune response all associated activated genes are shown, while for the rest, the top 10-15 genes were picked based on the magnitude of fold change expression. The top 10 commonly downregulated genes in I and S with the highest fold change in expression are also shown.

We next compared the immune induced response of the fat body to the 252 ‘core’ genes found in averaged-out whole-body reaction (17). We observed only minimal overlap in the infection-induced upregulated genes in the fat body and the whole-body datasets and almost no commonality in the cohorts of downregulated genes. There were only 17 genes common in both S and I fat body and ‘core’, with most of them being AMP genes. Further, there were a few genes only common between infection (I) and ‘core’ which also mainly included AMPs, while in comparison to sterile injury (S) more immune-related genes were included (**Figures 2D, E**). Looking at the 30 most abundantly expressed upregulated genes on infection, we found some important immune effectors including AMPs and IMPPP and many ribosomal protein genes (**Figure 2F**). This indicates the importance of a highly upregulated translation machinery for the functionality of fat body against bacterial invaders. A considerable portion of regulated genes overlaps between both datasets from the fat body (sterile injury and infection, **Figure 2D**). These include nearly 377 upregulated genes, which could represent the ‘core’ gene set induced in immune-related stress in the fat body. GO term analysis of these central genes, as well as those ones specifically regulated in either treatment, indicated most genes were associated with similar processes. We provide a schematic summary of the processes upregulated and the percentage of genes activated in each process in S and I fat bodies (**Figure 3A**) and show the fold change in expression values for the most highly expressed genes for these processes (**Figure 3B**). Apart from the classical innate immune response, the most prominent process is translation with nearly 70 regulated genes. Most regulated genes are upregulated, which implies higher transcriptional/translational activity. Interestingly, GO terms such as proteasomal machinery, ubiquitin-based protease activity, and protein export are also enriched. We also observed terms related to increased oxidation-reduction processes, which are already known to be crucial for fighting infection or any stress. Oxidative phosphorylation-related GO terms are also enhanced, which points to a metabolically active fat body that requires much energy. We also see a small number of metabolic genes included in processes like lipid homeostasis, sugar transport, glycogen metabolism, and fatty acid degradation. The downregulated genes did not yield significant GO terms.

### Infection induced transcriptional changes in hemocytes are distinct from those observed in whole body data sets

**Figure 4:**
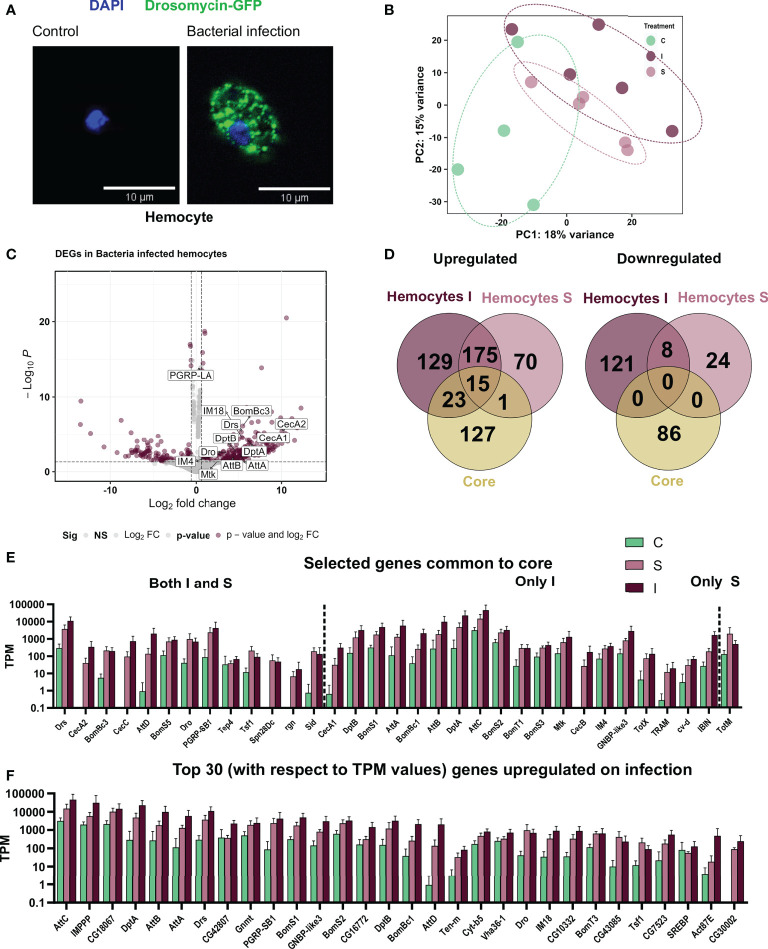
Responses of adult hemocytes to systemic infection and to sterile injury. **(A)** Hemocytes from the reporter line *Drosomycin-GFP* left untreated (left) and after infection with *B. subtilis* (right). The nuclei are stained with DAPI. **(B)** Principal Component Analysis plot showing the first two principal components for the RNA-seq analysis of control (C), sterile injury(S), and bacteria infected (I) adult hemocytes. **(C)** Volcano plot depicting the differentially expressed genes in bacteria infected hemocytes as compared to untreated control hemocytes. The genes passing the threshold (adjusted p-value < 0.05 and log_2_fold change > 0.6) were considered differentially expressed and are marked in maroon and the rest of the genes are marked in gray. Some of the differentially expressed AMPs are labeled in the graph. **(D)** Venn diagram analysis of the common number of regulated genes in the bacteria infected (I) and sterile injury (S) hemocytes (each compared to untreated controls) and the ‘core’ genes from the whole body are shown separately for the upregulated and downregulated genes. **(E)** Transcript per Million (TPM) values are shown for selected genes that were commonly regulated between the full body ‘core’ dataset and the bacteria infected (I) and sterile injury (S) hemocyte data sets. **(F)** TPM values are shown for the top 30 upregulated genes, i.e., the DEGs with the highest abundance values in terms of read counts, in bacteria infected hemocytes.

As for the fat bodies, we also could confirm the induced expression of AMPs in hemocytes upon infection using the AMP reporter lines (**Figure 4A**) and further identified this in the transcriptomic analysis (**Figure 4C**). The principal component analysis of the hemocyte samples showed a clustering based on their infection state (**Figure 4B**). We observe that samples from sterile wounding and from bacterial infection are closer to each other than unhandled control samples. Comparing treated hemocytes with controls, we identified 469 (341 upregulated, 128 downregulated) genes regulated in bacterial infection and 291 (260 upregulated and 31 downregulated) in sterile injury (**Figure 4D**). We further compared the immune-stress induced response of hemocytes to the whole-body ‘core’ immune-related genes. There was, as seen for the fat body, no significant commonality in the downregulated genes, while for activated genes, the small number of common genes mainly included AMP genes (especially for the bacterial infection treatment) and, in addition, a few genes involved in immune response pathways (**Figures 4E, F**)

### Functionally relevant processes activated in hemocytes upon infection

**Figure 5:**
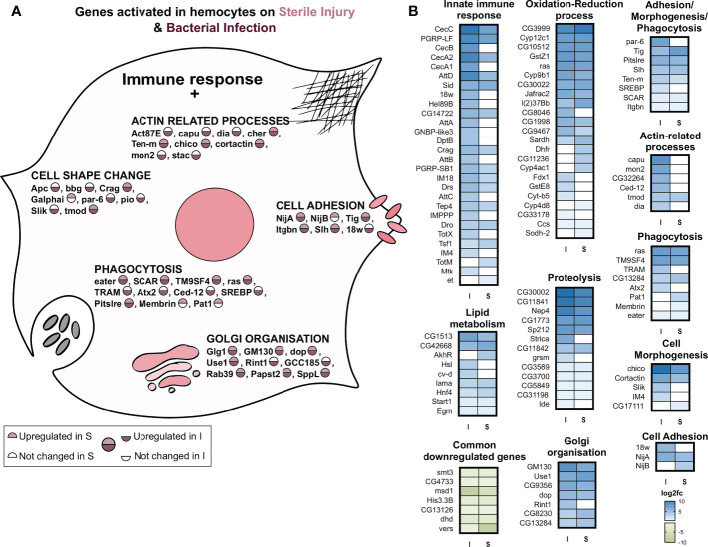
GO term analysis of the processes that are regulated in response to infection or sterile injury in adult hemocytes. **(A)** Summary of the processes activated in addition to the classic antibacterial response in hemocytes to bacterial infection (I) or sterile injury (S). For each process, selected genes are shown with the circle depicting their activation in I and S. As shown in the scale below, the upper half of the circle shows the upregulation in S, where no color means no change in expression while pink shows upregulation. The lower half shows the same information for I, with no color for no expression change and maroon for upregulation. **(B)** The log_2_ fold changes of genes associated with the GO terms of the processes shown A are plotted as heatmaps for bacterial infection (I) and sterile injury (S) hemocytes. The commonly downregulated genes in I and S are also shown.

We see a ‘central’ set of 190 genes commonly upregulated upon sterile injury and bacterial infection (**Figure 4D**). Considering both treatments, we found that the GO term with the highest statistical support is “immune response”. Although the number of genes was not large enough for a statistically relevant GO term analysis, we found some genes associated with relevant processes (**Figure 5**). Many genes were related to the oxidation-reduction process, as in the fat body, which might be essential for a highly active hemocyte to fulfill its tasks. Other genes we noticed included well-known and essential targets of the processes like actin cytoskeleton rearrangement, microtubule organization, cell morphogenesis, phagocytosis, cell migration, and adhesion. Some genes were also conspicuous that are related to metabolic changes, including lipid metabolism, Golgi organization, and proteolysis. The lipid homeostasis genes were also active in larval hemocytes on an infection or wound (33). This points to an important role of lipid homeostasis in hemocytes facing immune stress. On top of that, the active hemocytes show increased expression of genes associated with an increased breakdown of proteins, which could provide energy and metabolites. The downregulated genes in infected hemocytes have, presumably due to the low number, no significantly enriched GO terms.

One aim of this study was to gain a deeper understanding of the mechanisms in adult hemocytes that are induced after infection. To this end, we analyzed in more detail the top 30 expressed genes, i.e., those with probably the greatest importance, among the upregulated ones (**Figure 4F**). As expected, most of the induced antimicrobial peptides were among the most highly expressed genes in hemocytes after infection. *AttC* was the most highly expressed gene, followed by *baramicin A2* (IMPPP) and *bombardier* (CG18067). Moreover, among the most highly expressed genes were other *attacins*, *diptericins*, and *bomanins*, as well as *drosomycin*. Expression of these AMPs in hemocytes may also aid in killing ingested *Bacillus subtilis* in systemic infection experiments. Phagocytosis and killing of *B. subtilis* is a phylogenetically ancient process (37). Of note are genes that are not primarily associated with an immune response, but which may be particularly important, for example, for digesting ingested bacteria (*Vha36-1*). In addition, several genes that play central roles in the control of metabolism should be mentioned, with *Gnmt* and *SREBP* being particularly prominent. Comparing this list of most highly expressed genes induced by infection with those following sterile injury reveals significant differences. It is striking that, those AMPs that were upregulated in response to infection did not do so to the same extent after sterile injury.

### AMPs are the only commonly activated genes in the fat body, hemocytes, and the whole-body dataset on infection

Next, we compared the response in the two cell types following bacterial infection. Combining the samples from fat bodies and hemocytes in a PCA showed separate clusters for the two (**Supplementary** **Figure 2A**). Further, while PC1 mainly explained the differences between the two cell types, PC2 explained the differences between control and infected tissue. We further observed this by focusing on the top 50 genes associated with both treatments using PCA (**Supplementary** **Figure 2B**).

**Figure 6:**
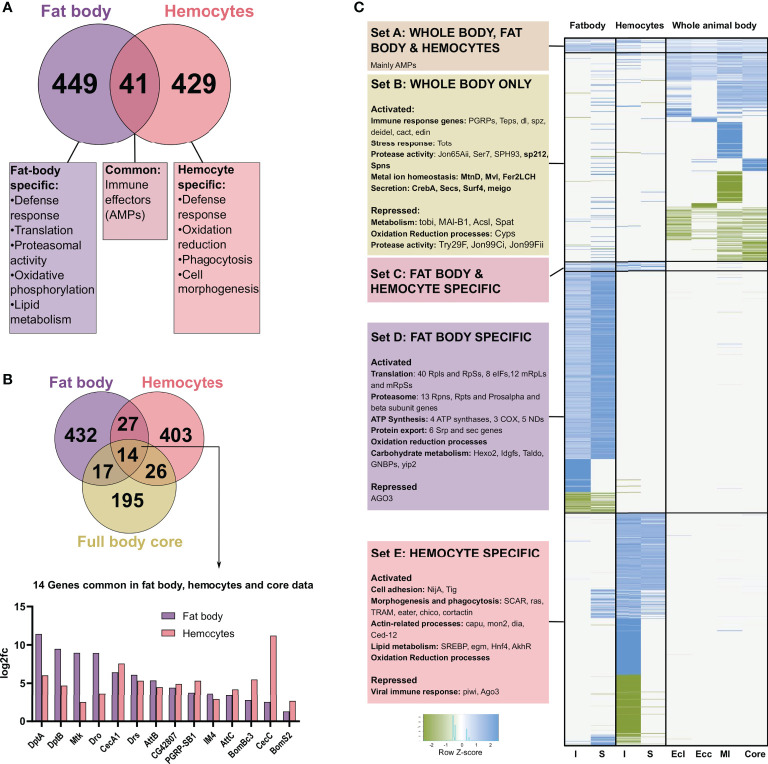
Comparison of the genes differentially expressed after infection and sterile injury, in fat bodies, hemocytes, and whole body data sets. **(A)** The number of commonly regulated genes in fat bodies and hemocytes on bacterial infection is shown with a summary of the common and distinct processes activated in each cell type. **(B)** Venn diagram showing the number of commonly regulated genes in bacteria infected fat bodies, hemocytes, and the ‘core’ from full body data (). The 14 genes commonly regulated in all three are shown at the bottom with the log_2_ fold changes in the expression in fat bodies and hemocytes. Most of these common genes are AMPs. **(C)** Heatmap showing the clustering of expression data from I and S fat body and hemocytes and the whole-body data for *E coli* (Ecl), *Ecc15* (Ecc), *M. luteus* (Ml), and the core. K-means clustering was performed on the genes differentially expressed in bacteria infected fat bodies, hemocytes, and full body data from Ecl, Ecc, Ml, and core based on the absence or presence of regulation of a gene. The scaled values for the log_2_ fold change are plotted here for the different clusters. The data was further divided into biologically relevant sets of clusters. Set A consists of genes differentially expressed in all the treatments and consisted mainly of AMPs, while Set C is only differentially expressed in fat bodies and hemocytes. Set B, D, and E are the sample-type specific clusters for whole body, fat body, and hemocytes respectively with some relevant categories and example genes shown for each set.

Comparing the lists of genes regulated in fat bodies and hemocytes from infected animals, we observed about 10% (41) of genes shared between both, and a major category in this group of commonly regulated genes are immune effector or AMP genes (**Figure 6A**). Focusing on specific genes, we observed distinct processes regulated in the two cell types. A summary of the processes specific to each cell type is shown (**Figure 6A**). While the fat body exhibit responses associated with metabolic and energetic changes like protein turnover in the form of translation, proteasomal activity and export, lipid metabolism and oxidative phosphorylation, hemocytes show responses associated with cell morphogenetic changes like actin-related processes, cell adhesion, and phagocytosis. One common process between the two is oxidation-reduction, which is functional through very different genes.

We next compared the bacterial infection-induced response of the fat body and hemocytes to the ‘core’ genes found in averaged-out whole-body responses to different bacterial infections (33). Interestingly, we see only 14 genes common between the three, out of which 12 were AMP genes and one a PGRP gene (**Figure 6B**). Subsequently, we investigated if the commonality between whole body and tissue-specific data changes if we look at information from infection experiments with specific bacteria instead. We picked *M. luteus*, *E. coli*, and *E. cc15* (17). This is a collection of gram-positive and negative bacteria, where patterns of host survival and pathogen clearance were similar to the *Bacillus subtilis* (gram-positive bacterium with DAP-type peptidoglycan) infection used in the current study. Based on the presence or absence of differential expression, we clustered the collection of genes differentially expressed in the animals infected with one of these three bacteria, the core or bacteria-infected fat body and hemocytes, and plotted the scaled fold change values (**Figure 6C**). We observed a small cluster expressed in all of them, and it contained only AMPs (**Figure 6C**-SetA). Quantitatively, only 10-20% of the genes expressed in full-body data (either core or specific pathogen) are also regulated in the fat body or hemocyte samples (**Supplementary Figure 2C**), respectively. Here, 30-50% in the fat body and 40-60% in hemocytes data sets are AMP genes (**Supplementary Figure 2D**). Another small cluster is Set C in **Figure 6C**, with the genes shared between the fat body and hemocytes. However, the most prominent and highly distinct clusters are specific to the cell types (Set D and E).

## Discussion

We systematically examined the transcriptional responses of isolated *Drosophila* immune organs to systemic infection and to sterile injury. Comparing both professional immune organs, fat body, and hemocytes, revealed a surprisingly low concordance of transcriptional profiles. This strongly divergent transcriptional response of fat bodies and hemocytes to infection and sterile injury was surprising especially considering that both organs use the same signalling systems for pathogen recognition (1, 16). The cohort of genes upregulated after infection in both fat body and hemocytes consisted mainly of immune effector-encoding genes. A closer look at those genes that were regulated in only one of the two organs shows that they represent immune-associated aspects that fit well with the specific tasks of humoral and cellular immunity, respectively. For hemocytes, these are mainly GO terms associated with phagocytosis of particles in the narrower and broader sense. While phagocytosis as a GO term directly fits the expectations, processes including cell adhesion, cell shape changes, actin-related processes, and Golgi organization are also linked to phagocytosis. *Bacillus subtilis* is usually not considered as a pathogen, but we observed a pathogenic potential in systemic infection. While the mechanism of infection is not well studied for *Bacillus subtilis*, phagocytosis would serve as an essential process for the functionality of hemocytes against any invading bacterium. In contrast, responses of the fat body focus on the synthesis and export of antimicrobial peptides. Here, appropriate GO terms such as protein transport and translation are foremost. However, GO terms such as proteasome and fatty acid degradation represent secondary effects providing metabolites and energy, respectively. The increased mitochondrial activity, which enables more ATP production, could also be in the same context.

We observed minimal overlap when comparing the already available whole-body data (17) to our tissue-specific dataset from the two major immune-responsive cell types. Several reasons might account for this. On the one hand, it is possible that there are significant differences in responses to infections with different pathogens, an aspect that is certainly important (38, 39). On the other hand, these differences may also represent secondary effects that could be explained by different degrees of infection events and responses caused by them (40, 41). We assume that the whole-body transcriptomic signatures in response to infection are neither dominated by the fat body responses nor by those of the hemocytes. Instead, other organs that show only secondary immune-related responses might dominate this response. Our results support this hypothesis because specific immune responses and sterile injury shared far more than 50% of commonly upregulated genes. This large overlap is seen both in the fat body and hemocytes implying that a large proportion of the infection-induced response in these professional immune organs is part of a core response launched in any infection of a certain intensity. Recent transcriptome studies focusing on the immune response of the *Drosophila* fat body further this hypothesis (42). In these studies, infections with gram-positive or gram-negative bacteria resulted in approximately 50% concordantly induced transcripts (42). Larval hemocytes responded in a similar fashion to different types of infection (33). Thus, it seems that the core response of the insect immune system is essentially limited to antimicrobial effectors, among which antimicrobial peptides are most important. The immune responses of individual immunocompetent organs reveal a more comprehensive core that considers the specific requirements of the organs in question. This organ-specific core can thus be identified as the overlap in induced transcript changes of sterile injury and specific immune responses. However, a technical problem arises in infection experiments with living microorganisms, often complicating such comparisons. Here, the variance in such experiments is higher than, for example, in sterile injury. This high variance in infection experiments might depend on inter-experimental variation in the infection procedure, but, and this is more likely, be due to stochastic processes inherent in the infection process (36).

Looking at how the fat body reacts to an infection and comparing this with the reaction to a sterile injury, the overlaps are, as already described, extremely large. On the one hand, there is the activation of the classical immune response, with a focus on the expression of antimicrobial peptide genes. The fat body shows significant modifications in the metabolic, cellular, and gene expression machinery. The most prominent process we observed along with the innate immune response is translation, which is highly active in all steps, including the basic setup of ribosomal proteins and initiation and elongation factors. Protein production is an energy-consuming process, though such high levels of translation can allow the fat body cells to reprogram their proteome and provide them with the plasticity to change and adapt to cope with the stress in this metabolically active phase. This protein production machinery could further help deliver AMPs into the hemolymph stream, a known fat body function. An increased protein export also supplements this role. Additionally, we see a substantial activation of proteasome and ubiquitin processes, which degrade misfolded proteins and act as quality control. It could be to ensure the quality of the highly active translational machinery or to hydrolyze the unused or unrequired proteins and augment the proteome reprogramming. The enhanced activity is a characteristic of cells with high secretory capacities such as insulin-producing and releasing cells (43, 44). Another possibility could be the removal of oxidized, and hence misfolded or nonfunctional, proteins produced as a side effect of a high ROS environment, a role proteasomes perform in macrophages (Gieche et al., 2001). We also see an enriched cellular oxidative phosphorylation, which could help with the energy requirements of translation and proteasome in the context of the immune response. All these processes, along with some carbohydrate metabolic functions including glycogen metabolism, sugar transport, and fatty acid beta-oxidation, indicate a highly active transcriptional landscape, which would be expected of this immuno-metabolic tissue. Thus, this usage of energy stores in fat body cells for the sake of immunity follows a well-accepted hypothesis of resource allocation during infection (13, 45, 46).

In the bacteria-infected fat body samples, we observed a high variability between the replicates. It is possible that injection with a capillary and delivering defined volumes would have reduced this high variability. Alternatively, a biological reason could also be considered. A recent study showed a high degree of stochasticity in *Drosophila* systemic infections (36). Furthermore, our results also support this second possibility, as all individual experiments induced strong activation of the core antimicrobial response. This is particularly evident from the expression values of AMPs in the different biological replicates of bacteria-infected and injured individuals’ fat bodies. All replicates show a consistently high expression of the AMPs in response to infection and injury, whereby far more genes are induced in infections than in sterile injury. For a bacterial infection, the AMPs are an essential immediate requirement to facilitate quick removal of the microbes and survival of the organism. In sterile injury, the focus of the tissue is the proper healing of the wound and providing the body with an appropriate metabolic environment to deal with the stress. Thus, they can invest less in AMP production and more in the rest of the processes.

In contrast to the study situation, we have regarding the immune response of the adult fat body, corresponding studies for adult hemocytes are not available so far. The main reason for the lack of comparative organ-focused studies of the immune response in adult *Drosophila* so far is technical. Hemocytes of adults are difficult to isolate in sufficient quantity making their analysis demanding (47). To circumvent this problem, we had to include appropriate amplification steps in the procedure (24) allowing us to perform reliable studies with less than hundred cells per biological replicate. Hemocytes react to an infection with bacteria and they orchestrate parts of the immune response, but they show no expansion in response to an infection (47), a finding that contrasts earlier studies that described an adult hematopoetic organ in *Drosophila* (48). We observed a number of relevant genes among the list of the top 30 genes with the highest transcript levels in response to infection. Besides the expected antimicrobial peptides, the high expression of *bombardier* is of note, as it has recently been identified as a hemolymph protein that enables delivery of bomanins and therewith enabling their anti-fungal activity (49). We could therewith show that hemocytes are an important source of this highly important hemolymph protein. Moreover, the high expression of the vacuolar proton transporter gene *Vha36-1* has to be mentioned. It is involved in the acidification of lysosomes, which is an essential task for the inactivation of ingested microbes (50). We also found transferin1 (*Tsf1*) as being among the genes with the highest expression further highlighting this arm of the immune system as an iron scavenger thus preventing microbial growth (51). Again, here, we found that hemocytes are an important source of this highly important hemolymph protein. Finally, we also found unexpected genes whose products are important regulators of metabolism with *Gnmt* and SREBP as being the most important ones that had yet not been associated with hemocytes. *Gnmt* is a central regulator of the S-adenosyl-methionine (SAM) metabolism that is induced upon infection and damage, but which has up to now mostly been associated with the fat body (52). SREBP on the other hand is a central regulator of fat and cholesterol metabolism and might be operative in fueling the energy-demanding immune response (53). Focusing on the most strongly expressed genes in response to infection seems justified especially for the comparison with sterile injury. The simple comparison of the up-regulated genes revealed large similarities, suggesting that the responses are very similar. However, focusing only on the most highly expressed genes it became apparent that the top expressed genes are not activated to the same extent following sterile injury. This means that this quantitative component can be particularly important for the classification of the biological significance of an immune or stress response.

Observing the activation of a hemocyte by an immune stressor, we see the upregulation of many immune effectors at high levels, including most AMPs. The number of AMPs expressed, and, in some cases, the expression level is higher than in the fat body. It could indicate that adult hemocytes also contribute to the release of AMPs into the hemolymph, as speculated in larval plasmatocytes. However, it would be intriguing if hemocytes perform this role in adults, despite their small numbers. This local activity of hemocytes can also possibly facilitate transmission of ‘stress’ or ‘infection’ information from the hemocytes to the surrounding tissues (47). It is crucial to point out that we also saw the expression of a few genes that are involved in phagocytotic processes and interact with the Actin-related protein (Arp) 2/3 complex (54), a significant regulator of actin nucleation, polymerization, and lamellopodia formation in different cell types. The most relevant of these is the *SCAR* gene which forms a complex with *WAVE* to control actin rearrangement (55). Other relevant and upregulated genes include *Cip4* (56) and *Cortactin* (54), both activators of the Arp2/3 pathway, and Diaphanous (57) that interacts antagonistically with Cip4 to maintain actin rearrangement homeostasis. This suggests that Wasp/WAVE-Arp2/3 pathway could play a critical role in cellular morphogenetic changes in hemocytes on infection. We also found many other genes known for these morphogenetic changes in other cell types or developmental stages in different roles, which could be functionally conserved in an activated hemocyte for similar processes.

One of the very few processes that are activated in both cell types in addition to the central immune responses is the regulation of redox homeostasis. Reactive oxygen species have different tasks, but the strategy of protecting one’s own cells from damage is important in many contexts. In hemocytes, the production of ROS is a mechanism that is closely associated with the antibacterial function of these cells. ROS primarily serve to damage or kill bacteria (58). Interestingly, there is also a regulation of ROS in the fat body, which further questions what roles it would perform in organs not directly infiltrated by bacteria. Here, it is more plausible that the massive increase in the fat cells’ metabolism leads to the production of substantial amounts of ROS that have to be dealt with. Here, it would be a simple reaction to cope with the high energy demands.

Taking together, the response of hemocytes substantially differs from the fat body’s response. In hemocytes, besides the core immune response, mainly processes associated with phagocytosis are activated. In contrast to the fat body, the hemocyte could not use internal energy stores to fuel its activities. Thus, the enhanced lipid metabolic processes in hemocytes are highly interesting and require future research efforts. In the fat body, we have a completely different situation, where this immunocompetent organ has massive energy resources and uses this treasure to enable an effective immune response. Moreover, the significant responses in the fat body focus on enabling the production and release of antimicrobial peptides. For the overall immune response seen in the whole-body experiments, both organs mainly contribute to the AMPs, but most of the other transcriptomic signatures might come from different organs as a secondary response to the infection in combination with the response to the intense immune reaction.

## Data availability statement

The data presented in the study are deposited in the GEO repository, accession number GSE209960.

## Author contributions

VV and TR: Conceptualization. VV and SK: Investigation. All authors: Writing—review and editing. All authors contributed to the article and approved the submitted version.

## Funding

This work was funded by Kiel University as part of the International Max Planck Research School for Evolutionary Biology and the German Research Foundation (DFG) as part of the CRC 1182 (project C2) and for INST 257/591-1 FUGG.

## Acknowledgments

We would like to thank Britta Laubenstein and Christiane Sandberg for their excellent technical assistance. In addition, we would like to thank Daria Siekhaus (Klosterneuburg, Austria) and the Bloomington Drosophila Stock Center (Indiana, USA) for flies.

## Conflict of interest

The authors declare that the research was conducted in the absence of any commercial or financial relationships that could be construed as a potential conflict of interest.

## Publisher’s note

All claims expressed in this article are solely those of the authors and do not necessarily represent those of their affiliated organizations, or those of the publisher, the editors and the reviewers. Any product that may be evaluated in this article, or claim that may be made by its manufacturer, is not guaranteed or endorsed by the publisher.
