# Supplementary material for: Hemocytes and fat body cells, the only professional immune cell types in Drosophila, show strikingly different responses to systemic infections
Source: Front Immunol. 2022 Nov 23;13:1040510. doi: 10.3389/fimmu.2022.1040510 (PMC9726733; doi:10.3389/fimmu.2022.1040510)
Supplement: Supplementary file 2 [file DataSheet_1.docx]

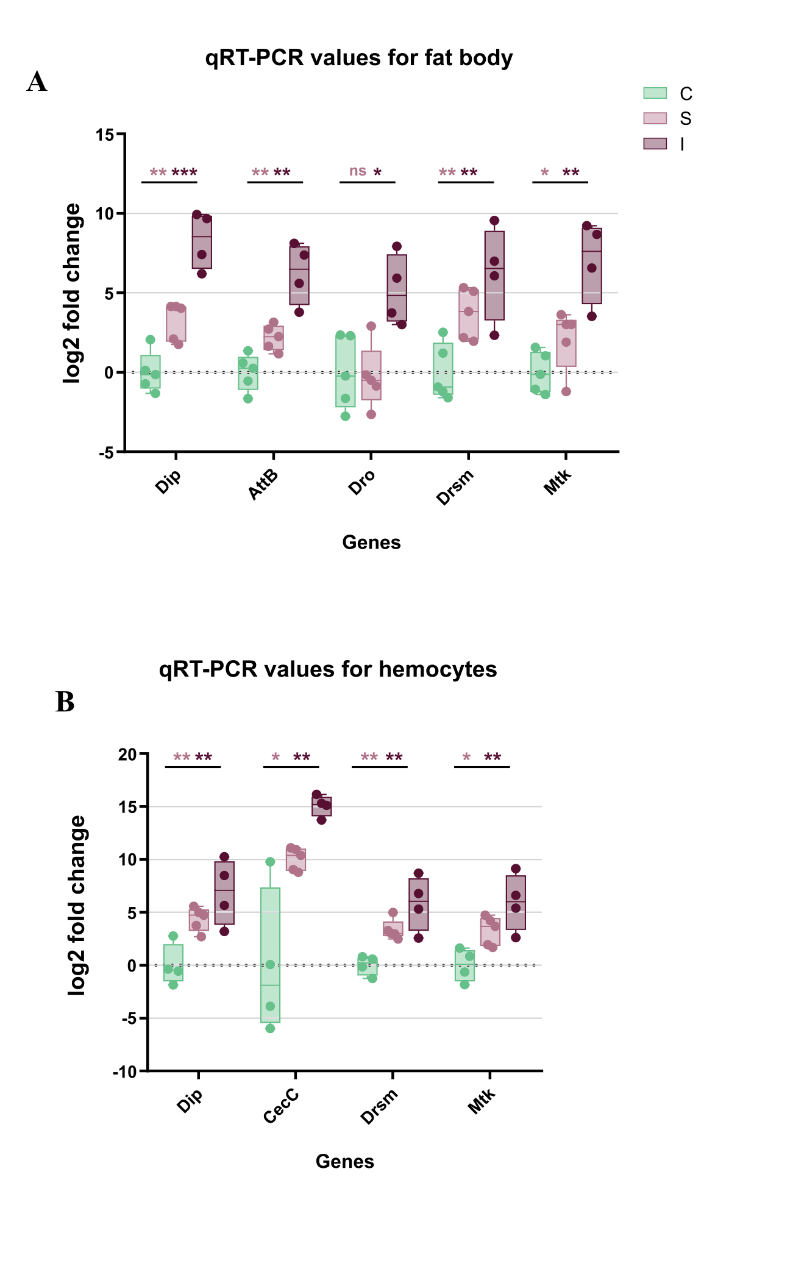
Supplementary Material

**Supplementary Figure 1:** qRT-PCR values to validate the functioning of the amplification system and RNA-sequencing **(A)** The log2 fold changes for selected AMP genes in the fat body upon infection and injury. 4-5 biological replicates were used for each treatment and RpL13 was used as a housekeeper gene for normalization. The log2fc values were calculated using the ∆∆Ct method. Statistical significance was calculated by performing multiple t-tests and using the two-stage linear step-up procedure of Benjamini, Krieger and Yekutieli to calculate the adjusted p-value. **(B)** The log2 fold changes for selected AMP genes in the hemocytes upon infection and injury. 4 biological replicates were used for each treatment and RpL32 was used as a housekeeper gene for normalization. The log2fc values were calculated using the ∆∆Ct method. Statistical significance was calculated by performing multiple t-tests and using the two-stage linear step-up procedure of Benjamini, Krieger and Yekutieli to calculate the adjusted p-value.


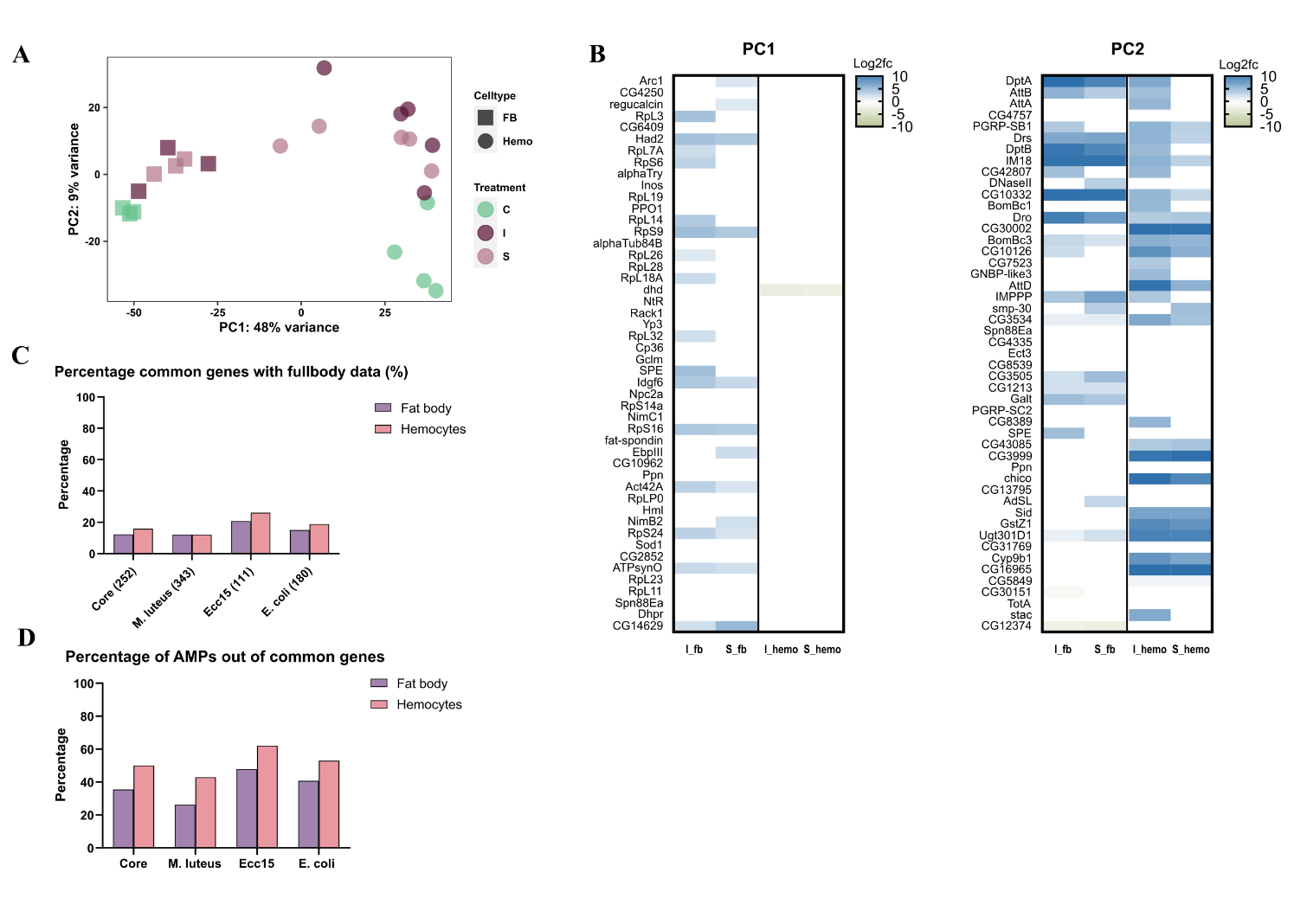


**Supplementary Figure 2:** Comparison of the induced transcriptional changes in response to infection and sterile injury. (A) PCA plot showing the first two principal components for the RNA-seq data of control (C), sterile injury (S), and bacteria-infected (I) hemocytes and fat bodies. (B) The top 50 genes contributing to PC1 and PC2 for the PCA plot are shown in A. We observed that PC1 explained more the differences between the two cell types and the top 50 genes contributing to PC1 are mainly the ones only expressed in fat body. PC2 explained more the differences between the different treatments and here the top 50 genes contain the ones expressed in both cell types with AMPs being a part of this list. (C) The percentage of commonly regulated genes in bacteria-infected fat body and hemocyte samples to the different full body bacterial infection treatments (from [17]). The number of genes regulated in each infection treatment is shown in brackets on the x-axis. (D) Percentage of AMPs out of the total common genes in C.

**Supplemental table 1:**

**Lists of differentially expressed genes (DEGs) in fat bodies and hemocytes in infection and sterile injury experiments.**
